# Dual regulation of receptor tyrosine kinase genes *EGFR* and *c-Met* by the tumor-suppressive *microRNA-23b/27b* cluster in bladder cancer

**DOI:** 10.3892/ijo.2014.2752

**Published:** 2014-11-14

**Authors:** TAKESHI CHIYOMARU, NAOHIKO SEKI, SATORU INOGUCHI, TOMOAKI ISHIHARA, HIROKO MATAKI, RYOSUKE MATSUSHITA, YUSUKE GOTO, RIKA NISHIKAWA, SHUICHI TATARANO, TOSHIHIKO ITESAKO, MASAYUKI NAKAGAWA, HIDEKI ENOKIDA

**Affiliations:** 1 Department of Urology, Graduate School of Medical and Dental Sciences, Kagoshima University, Kagoshima 890-8520, Japan; 2 Department of Pulmonary Medicine, Graduate School of Medical and Dental Sciences, Kagoshima University, Kagoshima 890-8520, Japan; 3 Department of Functional Genomics, Chiba University Graduate School of Medicine, Chiba 260-8670, Japan

**Keywords:** bladder cancer, microRNA, *miR-23b*, *miR-27b*, tumor suppressor, *EGFR*, *c-Met*

## Abstract

Recent clinical trials of chemotherapeutics for advanced bladder cancer (BC) have shown limited benefits. Therefore, new prognostic markers and more effective treatment strategies are required. One approach to achieve these goals is through the analysis of RNA networks. Our recent studies of microRNA (miRNA) expression signatures revealed that the *microRNA-23b/27b (miR-23b/27b)* cluster is frequently downregulated in various types of human cancers. However, the functional role of the *miR-23b/27b* cluster in BC cells is still unknown. Thus, the aim of the present study was to investigate the functional significance of the *miR-23b/27b* cluster and its regulated molecular targets, with an emphasis on its contributions to BC oncogenesis and metastasis. The expression levels of the *miR-23b/27b* cluster were significantly reduced in BC clinical specimens. Restoration of mature *miR-23b* or *miR-27b* miRNAs significantly inhibited cancer cell migration and invasion, suggesting that these clustered miRNAs function as tumor suppressors. Gene expression data and *in silico* analysis demonstrated that the genes coding for the epidermal growth factor receptor (*EGFR*) and hepatocyte growth factor receptor (*c-Met*) were potential targets of the *miR-23b/27b* cluster. Luciferase reporter assays and western blotting demonstrated that *EGFR* and *c-Met* receptor trypsine kinases were directly regulated by these clustered miRNAs. We conclude that the decreased expression of the tumor-suppressive *miR-23b/27b* cluster enhanced cancer cell proliferation, migration and invasion in BC through direct regulation of *EGFR* and *c-Met* signaling pathways. Our data on RNA networks regulated by tumor-suppressive *miR-23b/27b* provide new insights into the potential mechanisms of BC oncogenesis and metastasis.

## Introduction

In developed contries, bladder cancer (BC) is the fifth most commonly diagnosed tumor and the second most common cause of death in patients with genitourinary tract malignancies (1). BCs can be classified into two categories: non-muscle-invasive tumors and muscle-invasive tumors. The 5-year survival frequency for patients with non-muscle-invasive BC is close to 90%, whereas patients with muscle-invasive tumors have 5-year survival frequencies of ~60% (2). Patients with non-muscle-invasive BC tend to have a high rate of recurrence. Moreover, some patients are found to have muscle-invasive BC at recurrence (3). Recent clinical trials of chemotherapeutics for advanced BC have shown limited benefits. Therefore, new prognostic markers and more effective treatment strategies are required. One approach to achieve these goals is through the analysis of RNA networks.

Recent studies have demonstrated the importance of non-coding RNAs (ncRNAs). Participation of these RNAs in human diseases, including cancer, is now apparent (4). For example, microRNAs (miRNAs) are small ncRNA molecules (19–22 bases in length) that regulate protein-coding gene expression by repressing translation or cleaving RNA transcripts in a sequence-specific manner (5). Numerous recent studies have reported that miRNAs are aberrantly expressed in many human cancers. In fact, miRNAs play significant roles in the initiation, development and metastasis of human cancers (6).

Important new information has been gained through the analysis of the cancer-related miRNA networks. We have used our miRNA expression signatures to investigate several tumor-suppressive miRNAs and their regulated cancer pathways (7–11). Notably, some miRNAs are located in close proximity in the human genome; these are termed clustered miRNAs. We previously reported that *miR-1/133a*, *miR-29s*, *miR-143/145* and *miR-195/497* formed clusters and that these clusters function as tumor suppressors, targeting several oncogenic genes in human cancers, including BC (7,12–21).

Previously, our miRNA expression signatures revealed that *miR-23b/27b* clustered miRNAs were significantly reduced in several cancer tissues (8,9). Our deep sequencing miRNA signature of BC also annotated downregulation of *miR-23b* in cancer tissues (7). In contrast, Jin *et al* showed that the expression of *miR-23b* and *miR-27b* was highly upregulated in human breast cancer, and knockdown of these miRNAs substantially repressed breast cancer growth (22). Thus, the expression status of the *miR-23b/27b* cluster is not consistent among different types of cancers. Importantly, the functional roles of the *miR-23b/27b* cluster have not been fully investigated in BC.

The aim of the present study was to investigate the functional significance of *miR-23b/27b* clustered miRNAs and to identify the molecular targets regulated by these miRNAs in BC cells. We found that restoration of *miR-23b* or *miR-27b* mature miRNAs significantly inhibited cancer cell migration and invasion. Gene expression data and *in silico* analysis demonstrated that epidermal growth factor receptor (*EGFR*) and hepatocyte growth factor receptor (*c-Met*) were potential targets of the *miR-23b/27b* cluster. Elucidation of the cancer pathways and targets regulated by tumour-suppressive *miR-23b/27b* cluster will provide new insights into the potential mechanisms of oncogenesis and metastasis of BC.

## Materials and methods

### Clinical specimens

A total of 58 BC and 25 normal bladder specimens were collected from patients who underwent cystectomy or transurethral resection of bladder tumors (TUR-BT) at the Kagoshima University Hospital. The 25 normal bladder specimens were derived from patients without BC. Samples were processed and stored in RNAlater^®^ (Qiagen, Valencia, CA, USA) at −20°C until RNA extraction. The samples were staged in accordance with the tumor-node-metastasis classification system of the American Joint Committee on Cancer/Union Internationale Contre le Cancer (UICC), and they were histologically graded. Written informed consent was obtained from all patients and the present study was approved by the Bioethics Committee of Kagoshima University. The patients’ backgrounds and clinicopathological characteristics are summarized in Table I.

### Cell culture and RNA extraction

We used the human BC cell lines BOY and T24. BOY was established in our laboratory from a male Asian patient, 66-years old, who was diagnosed with stage III BC with lung metastasis. T24 was obtained from the ATCC. The cell lines were incubated in minimum essential medium (MEM) supplemented with 10% fetal bovine serum and maintained in a humidified incubator (5% CO_2_) at 37°C. Total RNA was extracted as previously described (23).

### Quantitative real-time RT-PCR

Stem-loop RT-PCR for *miR-23b* (P/N 000400; Applied Biosystems, Foster City, CA, USA) and *miR-27b* (P/N 000409; Applied Biosystems) were used to quantitate miRNAs according to previously published conditions (11). To normalize data for quantifying the miRNAs, we used *RNU48* (P/N 001006; Applied Biosystems). The δ-δ threshold count method was used to calculate the fold-change.

### Mature miRNA transfection

As previously described (11), the BC cell lines were transfected with Lipofectamine RNAiMAX transfection reagent (Invitrogen, Carlsbad, CA, USA) and Opti-MEM (Invitrogen) with 10 nM mature miRNA molecules. As the negative control, Pre-miR miRNA precursor (P/N AM17111; Applied Biosystems) was used in gain-of-function experiments.

### Cell proliferation, migration and invasion assays

Cell proliferation was determined using an XTT assay performed according to the manufacturer’s instructions. Cell migration activity was evaluated with a wound healing assay and cell invasion assays were done using modified Boyden chambers as previously described (23). All experiments were performed in triplicate.

### Putative miR-23b and miR-27b target gene pathway analysis and expression

To obtain putative miR-23b- and *miR-27b*-regulated genes, we used the TargetScan database (Release 6.2, http://www.targetscan.org/). To identify signaling pathways regulated by the *miR-23b/27b* cluster, *in silico* and gene expression data were analyzed in the Kyoto Encyclopedia of Genes and Genomics (KEGG) pathway categories using the GeneCodis program. We performed gene expression analyses for all candidate genes involved in each of the pathways identified by GeneCodis3 software analysis using microarray expression data. The data were approved by the Gene Expression Omnibus (GEO) and were assigned GEO accession numbers (GSE11783 and GSE31684). For microarray expression data, we examined 90 BCs and 6 normal bladder epithelium collected from patients, none of whom had been exposed to chemotherapy before surgery. The data were normalized and analyzed with GeneSpring (Agilent Technologies, Santa Clara, CA, USA). Statistical analyses were conducted using the Mann-Whitney U test.

### Western blotting

Three days after transfection, protein lysates (20 μg) were separated in NuPAGE on 4–12% Bis-Tris gels (Invitrogen) and transferred to polyvinylidene fluoride membranes as previously described (23). Antibodies against EGFR and c-Met were purchased from Cell Signaling Technology (Danvers, MA, USA). Antibodies against GAPDH were purchased from Chemicon (Temecula, CA, USA). Specific complexes were visualized with an echochemiluminescence (ECL) detection system (GE Healthcare, Little Chalfont, UK).

### Plasmid construction and dual-luciferase reporter assays

miRNA target sequences were inserted between the *Xho*I-*Pme*I restriction sites in the 3′-untranslated region (UTR) of the *hRluc* gene in the psiCHECK-2 vector (C8021; Promega, Madison, WI, USA). miRNA target sequences targeted by *miR-23b* and *miR-27b* are summarized in Table II.

T24 cells were transfected with 50 ng of the vector and 10 nM miRNA using Lipofectamine 2000 (Invitrogen). The activities of firefly and *Renilla* luciferases in cell lysates were determined with a dual-luciferase assay system (E1910; Promega). Normalized data were calculated as the ratio of *Renilla*/firefly luciferase activities.

### Statistical analysis

Relationships between 2 or 3 variables and numerical values were analyzed using the Mann-Whitney U test or Bonferroni adjusted Mann-Whitney U test. Spearman’s rank test was used to evaluate the correlation between the expressions of *miR-23b* and *miR-27b*. Expert StatView, version 4 was used in these analyses.

## Results

### Expression levels of miR-23b/27b cluster in BC clinical specimens

**Figure 1:**
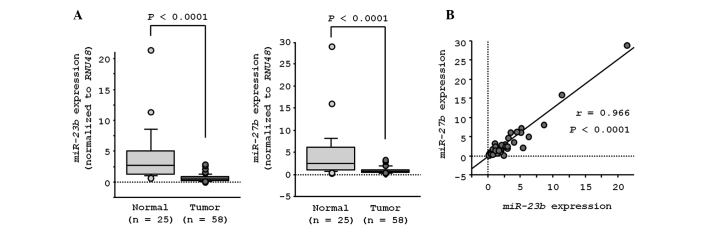
Expression levels of *miR-23b/27b* in clinical bladder specimens. (A) *miR-23b/27b* expression levels were significantly lower in 58 BC clinical specimens than in 25 normal bladder specimens. (B) The expression of *miR-23b* and *miR-27b* was positively correlated.

We evaluated the expression levels of *miR-23b* and *miR-27b* in BC tissues (n=58) and normal bladder specimens (n=25). The expression levels of *miR-23b* and *miR-27b* were significantly lower in tumor tissues than in corresponding non-cancerous tissues (both P<0.0001; Fig. 1A). Spearman’s rank test showed a positive correlation between the expression of *miR-23b* and that of *miR-27b* (r=0.966 and P<0.0001; Fig. 1B). These results suggested that *miR-23b* and *miR-27b* were significantly downregulated in BC and could represent putative tumor suppressors in BC.

### Effects of restoring miR-23b and miR-27b expression on cell proliferation, migration and invasion activities in cancer cell lines

**Figure 2:**
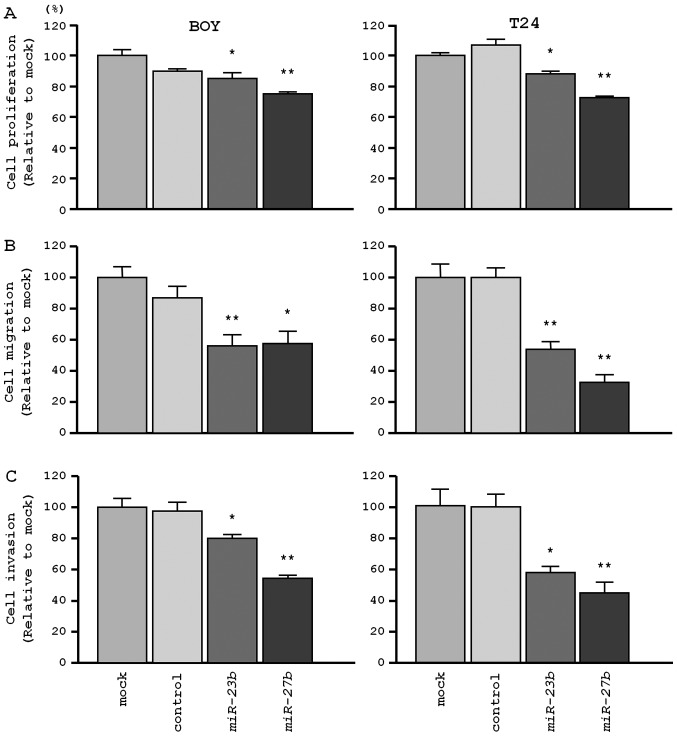
Effects of *miR-23b/27b* transfection into BC cell lines BOY and T24. (A) Cell proliferation determined by XTT assay. ^*^P<0.005. ^**^P<0.0001. (B) Cell migration activity determined with the wound healing assay. ^*^P<0.0005. ^**^P<0.0001. (C) Cell invasion activity determined with the Matrigel invasion assay. ^*^P<0.01. ^**^P<0.0001.

To examine the functional roles of *miR-23b* and *miR-27b*, we performed gain-of-function studies using miRNA transfection into BOY and T24 cells. XTT assays revealed significant inhibition of cell proliferation in BOY and T24 cells transfected with *miR-23b* and *miR-27b* in comparison with mock-transfected cells and control transfectants (BOY: P=0.0011 and P<0.0001, respectively; T24: P=0.0035 and P<0.0001, respectively) (Fig. 2A).

Moreover, wound healing assays demonstrated significant inhibition of cell migration was observed in BOY and T24 cells transfected with *miR-23b* and *miR-27b* (BOY: P<0.0001 and P=0.0001, respectively; T24: both P<0.0001) (Fig. 2B).

Similarly, *Matrigel* invasion assays revealed that transfection with these miRNAs reduced cell invasion. Indeed, the number of invading cells was significantly decreased in BOY and T24 cells transfected with *miR-23b* and *miR-27b* (BOY: P<0.0024 and P<0.0001, respectively; T24: P<0.0075 and P<0.0001, respectively) (Fig. 2C).

### Identification of targets pathways and genes regulated by the miR-23b/27b cluster in BC cells

**Figure 3:**
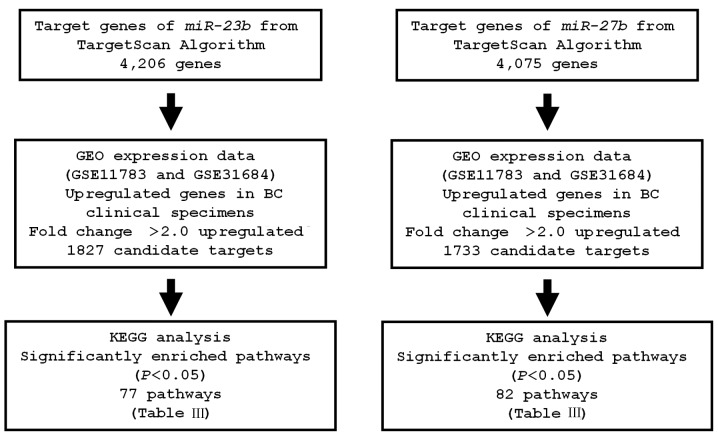
The strategy for selecting target pathways regulated by the *miR-23b/27b* cluster.

To gain further insight into the molecular mechanisms and pathways regulated by the tumor-suppressive *miR-23b/27b* cluster in BC, we performed a combination of gene expression and *in silico* analyses. The strategy for selecting *miR-23b/27b* cluster-regulated pathways is shown in Fig. 3.

The TargetScan program showed that 4206 and 4075 genes had putative target sites for *miR-23b* and *miR-27b*-, respectively, in their 3′-UTR regions. To confirm the expression levels of these genes in clinical BC tissues, GEO database (GEO accession number: GSE11783 and GSE31684) analysis was performed. The data showed that 1827 and 1733 genes were 2.0-fold or more upregulated in BC tissues compared to normal tissues for *miR-23b* and *miR-27b* target genes, respectively. KEGG analysis revealed that the top ‘pathway’ (greatest number of genes) was ‘Pathways in cancer’ (Table III). Several common putative target genes were included in this pathway (Table IV). We focused on *EGFR*, *RET* and *c-Met* genes that coded for tyrosine kinase receptors.

### EGFR and c-Met were regulated by miR-23b and miR-27b

**Figure 4:**
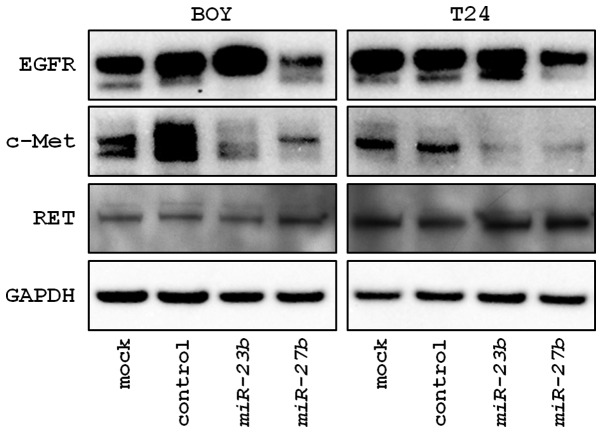
EGFR and c-Met protein expression levels were suppressed by *miR-23b/27b* transfection in BOY and T24 cells. Expression of EGFR, c-Met and RET protein as revealed by western blot analysis. GAPDH was used as a loading control.

We performed western blot analysis of BOY and T24 cells to investigate whether EGFR, RET and c-Met expression were downregulated by restoration of *miR-23b* and *miR-27b*. Expression of EGFR protein was significantly repressed in *miR-27b* transfectants in comparison with mock or miR-control transfectants (Fig. 4). The protein expression level of c-Met was significantly repressed in *miR-23b* and *miR-27b* transfectants ( Fig. 4). RET protein expression was not repressed in either *miR-23b* or *miR-27b* transfectants (Fig. 4).

### EGFR and c-Met were directly targeted by miR-23b and miR-27b

**Figure 5:**
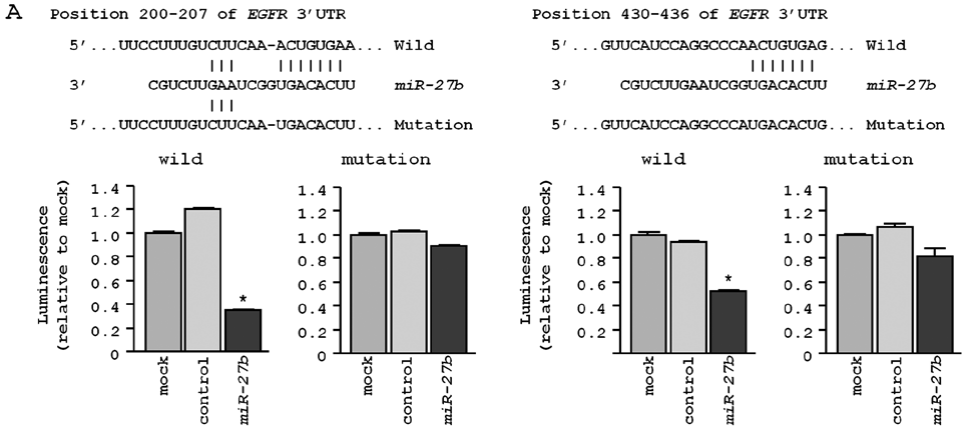

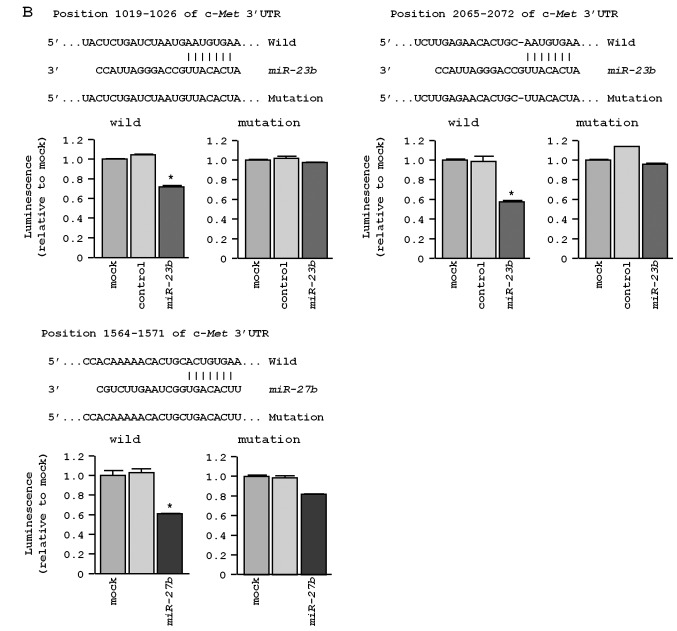
(A) Luciferase reporter assays using vectors encoding putative target sites in the 3′-UTR. T24 cells were transiently transfected with Pre-miR miRNA precursor or negative control, followed by transient transfection with wild-type 3′-UTR reporter plasmids or mutated 3′-UTR plasmids. *Renilla* luciferase activity was measured 24 h after transfection. The results are normalized to firefly luciferase values. ^*^P<0.0001. (B) Luciferase reporter assays using vectors encoding putative target sites in the *c-Met* 3′-UTR.

We performed a luciferase reporter assay in T24 to determine whether *EGFR* and *c-Met* had target sites for *miR-23b* and *miR-27b*. The TargetScan database predicted that two putative *miR-27b* binding sites existed in the 3′-UTR of *EGFR* (positions 200–207 and 430–436; Fig. 5A). The database showed that one putative *miR-23b* binding site existed in the 3′-UTR of *EGFR*. However, EGFR protein expression was not repressed in *miR-23b* transfectants (Fig. 4). Therefore, we performed a luciferase reporter assay to determine whether *EGFR* had target sites for *miR-27b*. The database also predicted that two putative *miR-23b* binding sites and one putative *miR-27b* binding site existed in the *c-Met* 3′-UTR (positions 1019–1026, 2065–2072 and 1564–1571, respectively; Fig. 5B). We used wild-type and mutant vectors encoding either the partial sequence of the 3′-UTRs of *EGFR* and *c-Met*, including the predicted *miR-23b* and *miR-27b* target sites.

We found that the luminescence intensity was significantly reduced by transfection of *miR-27b* with the wild-type vector carrying the 3′-UTR of *EGFR* (position 200–207: P<0.0001; position 430–436: P<0.0001; Fig. 5B), whereas transfection with a mutant vector showed no decrease in luminescence.

With regards to *c-Met*, the luminescence intensity was significantly reduced by transfection of *miR-23b* with vectors carrying a portion of the 3′-UTR of *c-Met* (position 1019–1026: P<0.0001; position 2065–2072: P<0.0001; Fig. 5B) and the luminescence intensity was also reduced by transfection of *miR-27b* with the vector carrying the 3′-UTR of *c-Met* (position 1564–1571: P<0.0001; Fig. 5B), whereas transfection with a mutant vector failed to decrease luminescence.

## Discussion

Aberrant expression of the *miR-23b/27b* cluster has been reported in several types of human cancers; however, the expression status varies according to the cancer type. Decreased expression of the *miR-23b/27b* cluster has been observed in castration-resistant prostate cancer (24) and drug-resistant Ehrlich ascites tumor (25). In contrast, upregulation of the *miR-23b/27b* cluster was reported in breast cancer (22) and chemoresistant ovarian cancer cells (26). In the present study, our data demonstrated that *miR-23b* and *miR-27b* expression was significantly downregulated in BC clinical specimens. Functional analysis demonstrated that restoration of *miR-23b* and *miR-27b* in BC cells inhibited cancer cell proliferation, migration and invasion. Those results suggest that the *miR-23b/27b* cluster functions as a tumor suppressor and may contribute to metastasis in BC. Of particular interest, our recent study showed that *miR-24-1*, which is located close to the *miR-23b/27b* cluster, was downregulated in BC tissues and functioned as a tumor suppressor targeting FOXM1 (27). Thus, our data suggest that the *miR-23b/27b* cluster, including *miR-24-1*, functions as a tumor suppressor and significantly contributes to BC oncogenesis and metastasis.

Next, we investigated the pathways/targets that were regulated by the tumor-suppressive *miR-23b/27b* cluster in BC cells. To identify tumor-suppressive, miRNA-regulated molecular pathways, we used a combination of expression data and *in silico* database analysis. Using this strategy, we have identified molecular targets and pathways in several types of cancer that are regulated by tumor-suppressive miRNAs, including BC (7–11). In the present study, ‘pathways in cancer’, the ‘MAPK signaling pathway’ and ‘cytokine-cytokine receptor interaction’ were significantly selected as candidate pathways regulated by the *miR-23b/27b* cluster in BC cells. Among these pathways, we focused attention on ‘pathways in cancer’ and searched for putative targets of *miR-23b/27b* regulation. We focused on tyrosine kinase receptors such as *EGFR*, *RET* and *c-Met* genes because molecularly targeted therapies aimed at inhibiting their activities have been developed recently for several types of cancer (28). Our results showed that *EGFR* was directly regulated by *miR-27b* and that *c-Met* was directly regulated by both *miR-23b* and *miR-27b*. Unfortunately, *RET* was not controlled by either miRNA.

*EGFR* is the cell-surface receptor for members of the epidermal growth factor family of extracellular protein ligands (29). Receptor activation initiates several signal transduction cascades, including the MAPK, Akt and JNK pathways, leading to DNA synthesis and cell proliferation (30). Previous studies showed that overexpression of EGFR occurred in BC and the expression level correlated with tumor grade, stage and survival (31–33).

Another receptor, *c-Met* (hepatocyte growth factor receptor) activates multiple signal transduction pathways such as those involving RAS, PI3K-Akt, STAT and β-catenin (34–37). The expression of c-Met and phosphorylated c-Met are positively correlated with tumor grade, stage, tumor size and survival of several types of cancers. It is likely that c-Met could be a promising therapeutic target in disease (38,39). The amplification frequency of c-Met is approximately 20% in patients who have acquired resistance to EGFR tyrosine kinase inhibitors (40). Therefore, inhibition of EGFR and c-Met and their associated signaling pathways could be a potent strategy for cancer therapy. Studies of dual tyrosine kinase inhibitors are underway (41). Several laboratories have shown that miRNAs directly inhibit EGFR or/and c-Met expression, such as *miR-7*, *miR-146a*, *miR-574-3p*, *miR-34a*, *miR-130a* and *miR-1/206* (42–47). In addition, a recent study demonstrated that *miR-27a* regulated both EGFR and c-Met in non-small cell lung cancer (48). In the present study, the *miR-23b/27b* cluster regulated EGFR and c-Met in BC cells. Therefore, inhibition of two separate tyrosine kinases via the tumor-suppressive *miR-23b/27b* cluster represents an attractive possibility in the development of new treatment options in cancer.

In conclusion, downregulation of the *miR-23b/27b* cluster is a frequent event in BC. Moreover, tumor-suppressive *miR-23b* and *miR-27b* directly regulated tyrosine kinase receptor genes *EGFR* and *c-Met*. Identification of molecular targets regulated by tumor-suppressive miRNAs will provide insights into the potential mechanisms of BC oncogenesis and metastasis, facilitating the development of novel therapeutic strategies for the treatment of BC.

## Figures and Tables

**Table I:** Patient characteristics.

Characteristics	Data
Bladder cancer	
Total number	58
Median age (range) (years)	71 (47–91)
Gender	
Male	45 (78%)
Female	13 (22%)
Pathological tumor stage	
pTis	2 (3%)
pTa	7 (12%)
pT1	10 (17%)
pT2	15 (26%)
pT3	7 (12%)
pT4	5 (9%)
Unknown	12 (21%)
Grade	
G1	2 (3%)
G2	29 (50%)
G3	21 (36%)
Unknown	6 (1%)
Operation	
Cystectomy	23 (40%)
TUR-BT	35 (60%)
Normal bladder epithelium	25

**Table II:** Insert 3′-UTR sequence of *EGFR* and *c-Met*.

Gene	Target sites	3′-UTR position		Sequence (5′-3′)
*EGFR*	miR-27b	200–207	Wild	gccaggaagtacttccacctcgggcacattttgggaagttgcattcctttgtcttcaa**actgtgaa**gcatttacagaaacgcatccagcaag aatattgtccctttgagcagaaatttatctttcaaagaggtatatttgaaaaaaaaaaaaagtatatgtgaggatttttattgattgg
			Mutation	gccaggaagtacttccacctcgggcacattttgggaagttgcattcctttgtcttcaa**tgacactt**gcatttacagaaacgcatccagcaag aatattgtccctttgagcagaaatttatctttcaaagaggtatatttgaaaaaaaaaaaaagtatatgtgaggatttttattgattgg
		430–436	Wild	ggatcttggagtttttcattgtcgctattgatttttacttcaatgggctcttccaacaaggaagaagcttgctggtagcacttgctaccctg agttcatccaggccca**actgtga**gcaaggagcacaagccacaagtcttccagaggatgcttgattccagtggttctgcttcaaggctt
			Mutation	ggatcttggagtttttcattgtcgctattgatttttacttcaatgggctcttccaacaaggaagaagcttgctggtagcacttgctaccctg agttcatccaggccca**tgacact**gcaaggagcacaagccacaagtcttccagaggatgcttgattccagtggttctgcttcaaggctt
*c-Met*	miR-23b	1019–1026	Wild	cattaagaaaatttgtatgaaataatttagtcatcatgaaatatttagttgtcatataaaaacccactgtttgagaatgatgctactctgat ctaatg**aatgtga**acatgtagatgttttgtgtgtatttttttaaatgaaaactcaaaataagacaagtaatttgttgataaatatttt
			Mutation	cattaagaaaatttgtatgaaataatttagtcatcatgaaatatttagttgtcatataaaaacccactgtttgagaatgatgctactctgat ctaatg**ttacact**tcatgtagatgttttgtgtgtatttttttaaatgaaaactcaaaataagacaagtaatttgttgataaatatttt
		2065–2072	Wild	gaactcggggaaacatcccatcaacaggactacacacttgtatatacattcttgagaacactgc**aatgtga**aaatcacgtttgctatttata aacttgtccttagattaatgtgtctggacagattgtgggagtaagtgattcttctaagaattagatacttgtcactgcctatacctgc
			Mutation	gaactcggggaaacatcccatcaacaggactacacacttgtatatacattcttgagaacactgc**ttacact**taatcacgtttgctatttata aacttgtccttagattaatgtgtctggacagattgtgggagtaagtgattcttctaagaattagatacttgtcactgcctatacctgc
	miR-27b	1564–1571	Wild	taactggttttgtcgacgtaaacatttaaagtgttatattttttataaaaatgtttatttttaatgatatgagaaaaattttgttaggccac aaaaacactgc**actgtgaa**cattttagaaaaggtatgtcagactgggattaatgacagcatgattttcaatgactgtaaattgcgata
			Mutation	taactggttttgtcgacgtaaacatttaaagtgttatattttttataaaaatgtttatttttaatgatatgagaaaaattttgttaggccac aaaaacactgc**tgacactt**cattttagaaaaggtatgtcagactgggattaatgacagcatgattttcaatgactgtaaattgcgata

**Table III:** Top 10 enriched pathways in *miR-23b* and *miR-27b*.

Annotations	No. of genes	P-value
*miR-23b*		
Pathways in cancer	50	5.09E-15
Neuroactive ligand-receptor interaction	39	5.52E-11
MAPK signaling pathway	24	4.60E-04
Cytokine-cytokine receptor interaction	23	8.97E-04
Endocytosis	22	6.98E-05
Focal adhesion	21	2.03E-04
Regulation of actin cytoskeleton	21	3.72E-04
Calcium signaling pathway	20	1.35E-04
Glutamatergic synapse	19	6.90E-06
Chemokine signaling pathway	19	5.79E-04
*miR-27b*		
Pathways in cancer	42	2.14E-10
MAPK signaling pathway	30	1.30E-06
Neuroactive ligand-receptor interaction	29	4.67E-06
Calcium signaling pathway	24	1.23E-06
Axon guidance	23	2.23E-08
Regulation of actin cytoskeleton	22	1.42E-04
Endocytosis	20	3.85E-04
Glutamatergic synapse	19	3.35E-06
Chemokine signaling pathway	18	1.34E-03
Cytokine-cytokine receptor interaction	18	2.10E-02

**Table IV:** *miR-23b/27b* common target genes highly expressed in bladder cancer.

Clinical BCs				
			
Change	P-value	Entrez gene ID	Symbol	Description
19.55	4.67E-05	7849	*PAX8*	Paired box 8
6.79	1.14E-04	1021	*CDK6*	Cyclin-dependent kinase 6
5.98	1.64E-04	1956	*EGFR*	Epidermal growth factor receptor
5.00	2.65E-04	2034	*EPAS1*	Endothelial PAS domain protein 1
4.74	1.21E-04	208	*AKT2*	v-akt murine thymoma viral oncogene homolog 2
4.60	5.91E-04	2246	*FGF1*	Fibroblast growth factor 1 (acidic)
4.20	8.23E-04	861	*RUNX1*	Runt-related transcription factor 1
3.96	3.16E-03	2250	*FGF5*	Fibroblast growth factor 5
3.91	3.75E-04	7170	*TPM3*	Tropomyosin 3
3.75	4.46E-04	4089	*SMAD4*	SMAD family member 4
3.75	5.59E-04	3918	*LAMC2*	Laminin, γ 2
3.42	7.80E-05	862	*RUNX1T1*	Runt-related transcription factor 1; translocated to, 1 (cyclin D-related)
3.31	6.45E-05	5979	*RET*	Ret proto-oncogene
3.09	1.75E-02	4286	*MITF*	Microphthalmia-associated transcription factor
2.87	8.69E-04	4233	*c-Met*	Met proto-oncogene
2.83	1.15E-02	2259	*FGF14*	Fibroblast growth factor 14
2.83	1.14E-04	3845	*KRAS*	Kirsten rat sarcoma viral oncogene homolog
2.68	2.32E-02	7188	*TRAF5*	TNF receptor-associated factor 5
2.63	2.73E-03	2932	*GSK3B*	Glycogen synthase kinase 3 β
2.60	1.82E-03	5594	*MAPK1*	Mitogen-activated protein kinase 1
2.40	1.48E-03	4193	*MDM2*	MDM2 oncogene, E3 ubiquitin protein ligase
2.37	1.40E-03	5579	*PRKCB*	Protein kinase C, β
